# Supplementary material for: Novel Synthesis of Choline-Based Amino Acid Ionic Liquids and Their Applications for Separating Asphalt from Carbonate Rocks
Source: Nanomaterials (Basel). 2019 Apr 1;9(4):504. doi: 10.3390/nano9040504 (PMC6523481; doi:10.3390/nano9040504)
Supplement: Supplementary file 1 [file nanomaterials-09-00504-s001.pdf]

Supporting Information

Novel Synthesis of Choline-Based Amino Acid Ionic Liquids and Their Applications for Separating Asphalt from Carbonate Rocks

Zisheng Zhang[1,2,3ζ] , Ning Kang[1,2ζ], Jingjing Zhou[1,2], Xingang Li[1,2,4], Lin He[1,2,4*], Hong Sui[1,2,4*]

1. School of Chemical Engineering and Technology, Tianjin University, Tianjin, PR China, 300072.
2. National Engineering Research Center of Distillation Technology, Tianjin, PR China, 300072.
3. Department of Chemical & Biological Engineering, University of Ottawa, 161 Louis Pasteur St., Ottawa, Canada K1N6N5.
4. Collaborative Innovation Center of Chemical Science and Engineering, Tianjin, PR China, 300072

E-mail: linhe@tju.edu.cn; suihong@tju.edu.cn; Tel.: 022-27404701

[ζ] These authors contributed equally to this work.

1. Characterizations of AAIL

1H NMR spectra of the synthesized AAIL of choline glycine (ChGly), choline serine (ChSer), choline histidine (ChHis), choline proline (ChPro), and choline phenylalanine (ChPhe) were recorded and shown in **Fig. S1~S5**. All the data are list in **Table S1**. According to the ratio of integration value of proton signals, the synthesized IL has a high purity.

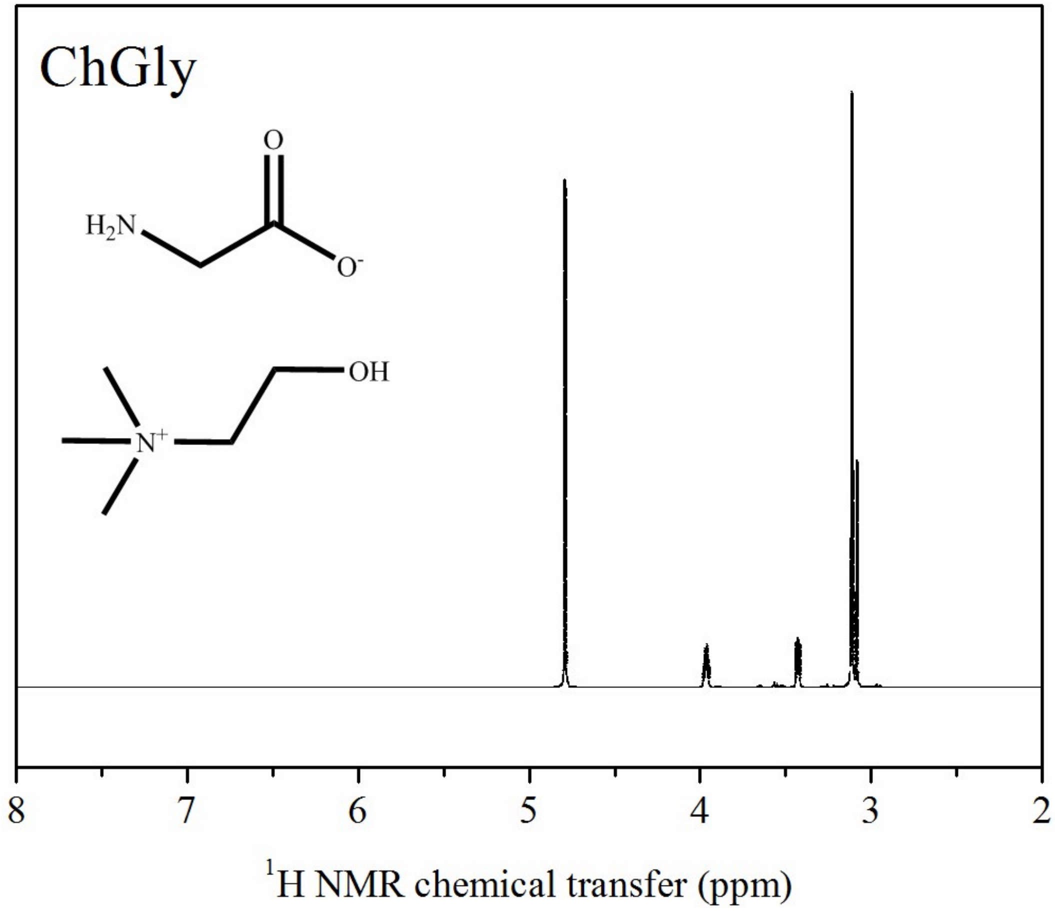

Figure S1. ^{1}H NMR spectra of ChGly. The solvent was D_2O as indicated by peaks near 4.79 ppm.

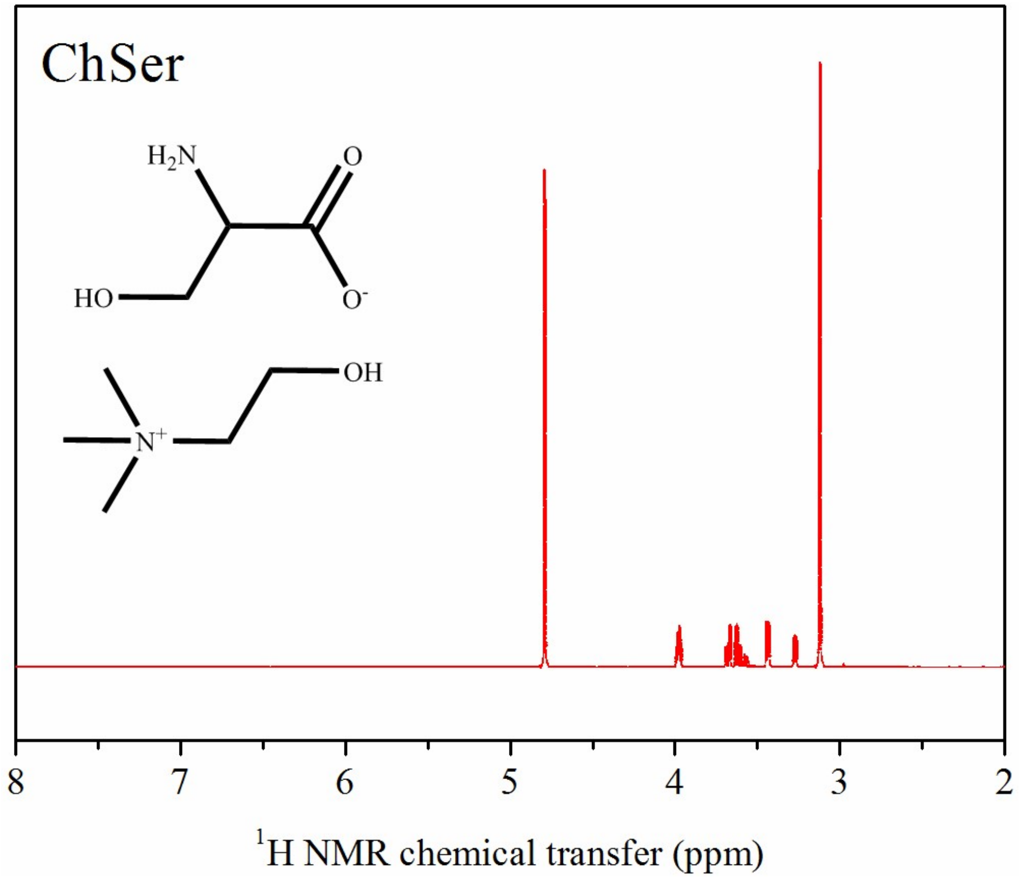

Figure S2. ^{1}H NMR spectra of ChSer. The solvent was D_2O as indicated by peaks near 4.79 ppm.

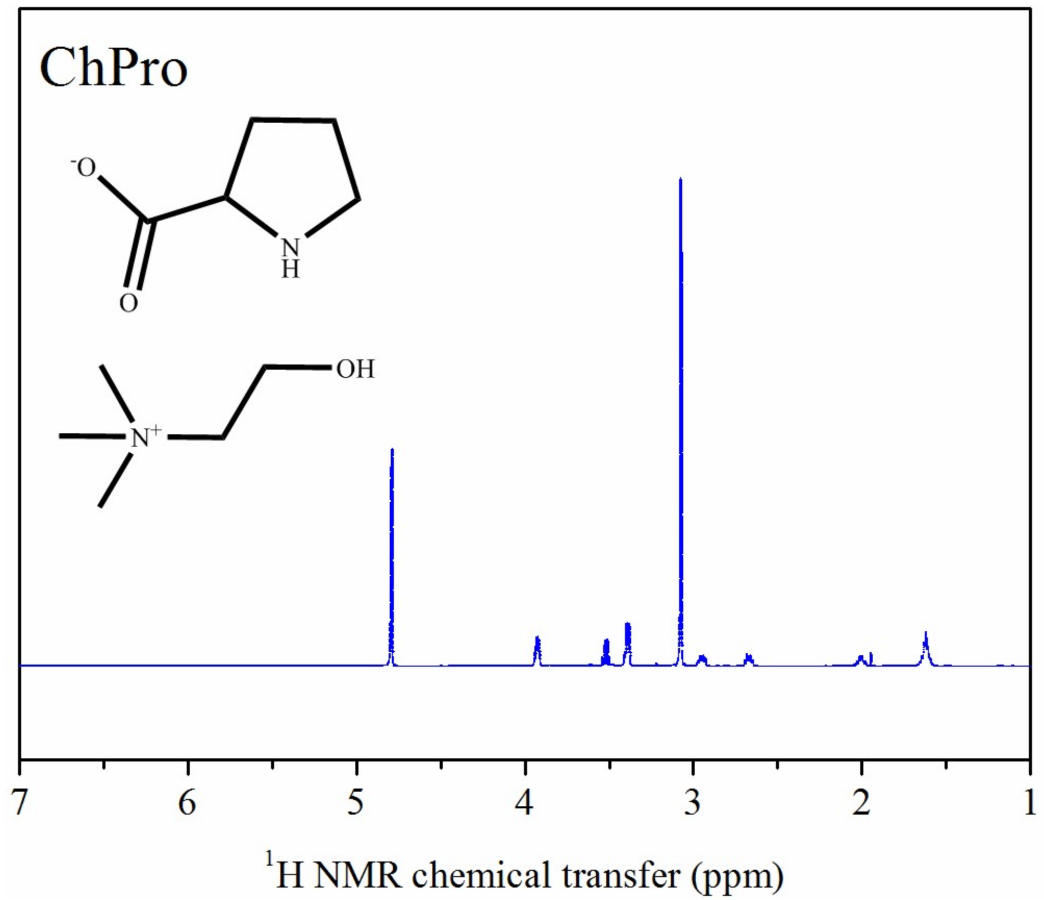

Figure S3. ^{1}H NMR spectra of ChPro. The solvent was D_2O as indicated by peaks near 4.79 ppm.

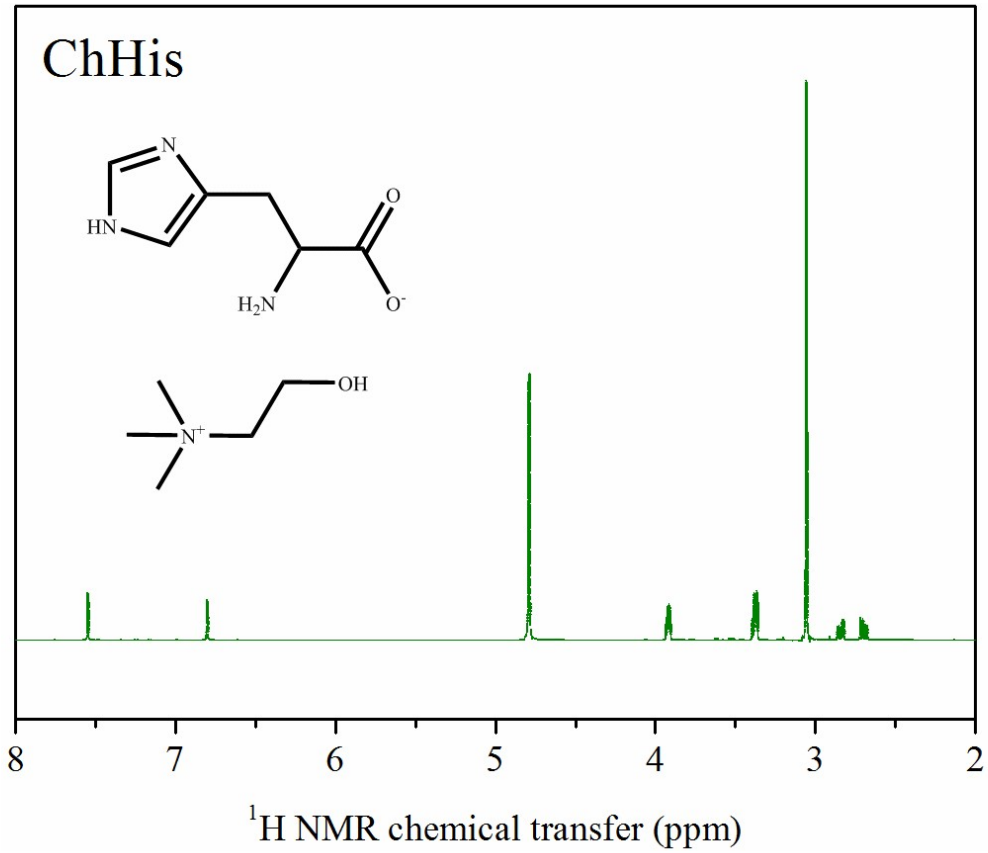

Figure S4. ^{1}H NMR spectra of ChHis. The solvent was D_2O as indicated by peaks near 4.79 ppm.

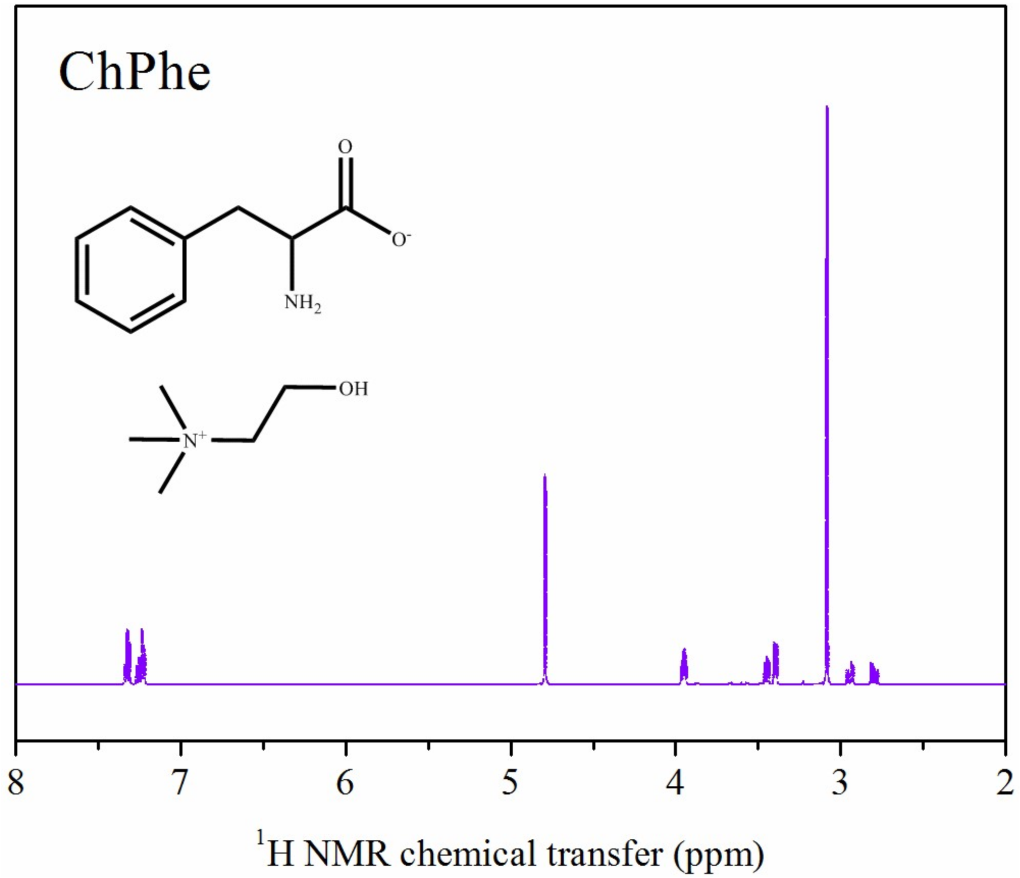

Figure S5. ^{1}H NMR spectra of ChPhe. The solvent was D_2O as indicated by peaks near 4.79 ppm.

Table S1. ^{1}H-NMR data of AAIL

	δ/ppm
ChGly	2.08 (s, 2H, CH2-N), 3.11 (s, 9H, CH3, CH3, CH3), 3.43 (m, 2H CH2), 3.96 (m, 2H, CH2)
ChSer	3.12(s, 9H,CH3, CH3, CH3), 3.27 (t, 1H, CH-N), 3.44 (t, 2H, CH2), 3.67 (d , 2H, CH2), 3.97 (m, 2H, CH2)
ChPro	1.62 (m, 3H, CH2, CH2), 2.00 (m, 1H, CH2), 2.67(m, 1H, CH2-N), 2.94 (m, 1H, CH2-N), 3.07 (s, 9H, CH3, CH3, CH3), 3.39 (m, 1H, CH-N), 3.51 (t, 2H, CH2), 3.92 (m, 2H, CH2)
ChHis	2.70 (m, 2H, CH2), 2.83 (m, 2H, CH2), 3.06 (s, 9H, CH3, CH3, CH3), 3.37 (t, 3H, CH2, CH-N), 3.91 (m, 2H CH2), 6.80 (s, 1H, =CH), 7.55 (s, 1H, =CH)
ChPhe	2.80 (m, 1H, CH2), 2.97 (m, 1H, CH2), 3.08 (s, 9H, CH3, CH3, CH3), 3.38 (m, 2H, CH2), 3.43 (m, 1H, CH-N), 3.95 (m, 2H, CH2), 7.23-7.32 (m, 5H, C6H5)

Based on DSC curves of AAIL (shown in Fig. S6), the glass transition temperatures were observed in the range from -83.17 °C to -49.71 °C for all the ILs. All AAILs were liquids at room temperature. The TGA curves of AAIL are shown in Fig. S7. The decomposition temperatures were calculated from the intersection of the baseline and the tangent line in the TGA curves. The decomposition temperatures ranked from 138 °C to 185 °C, which means these AAIL are thermally stable enough for IL-assisted solvent extraction test.

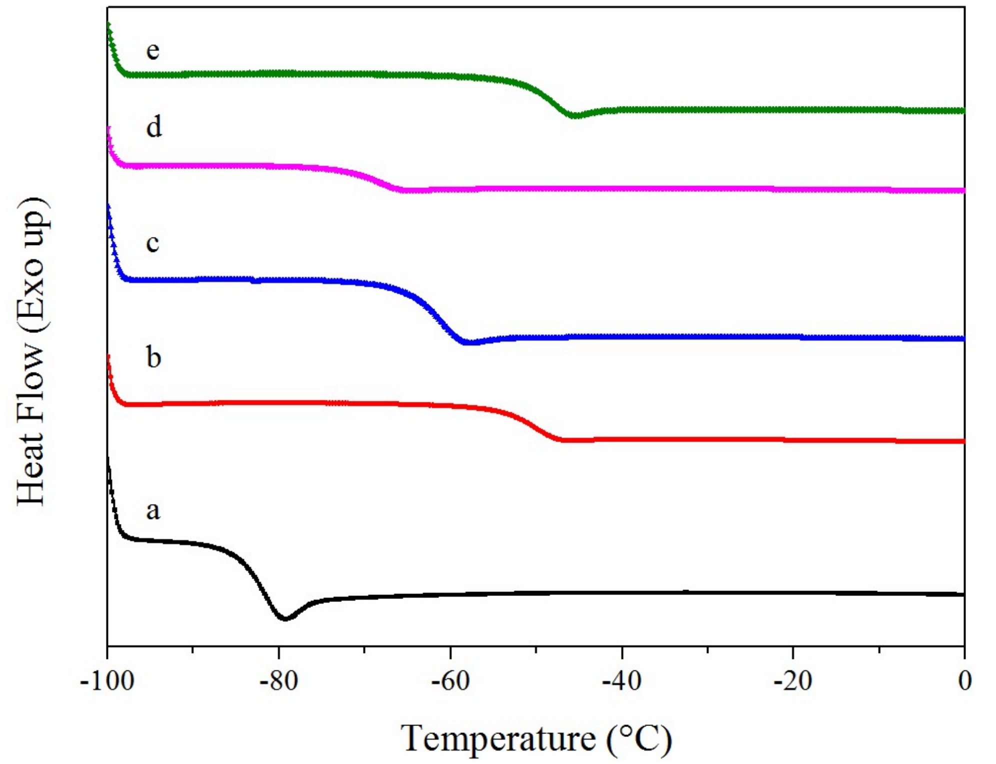

Figure S6. DSC heating traces for (a) ChGly, (b) ChHis, (c) ChSer, (d) ChPro and (e) ChPhe.

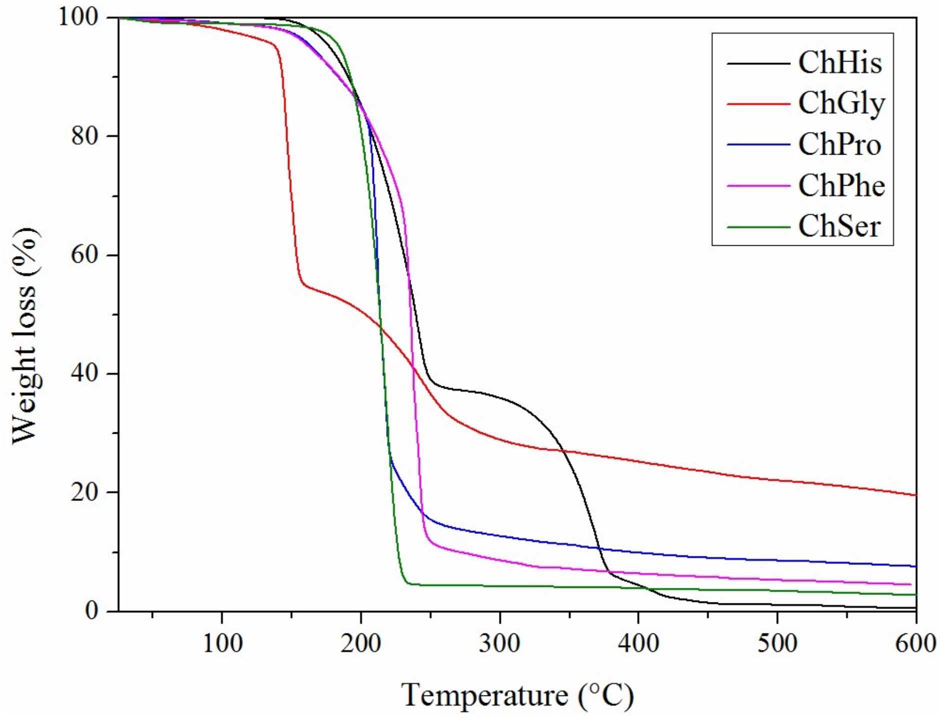

Figure S7. TGA curves for AAILs.
